# Degradable Alternatives to Single-Use Plastics: Mechanisms, Materials, and Strategies for Sustainable Polyolefin Replacement

**DOI:** 10.3390/molecules30214301

**Published:** 2025-11-05

**Authors:** Hamza Fakhrizada, Yaser Dahman

**Affiliations:** 1 Department of Electrical, Computer & Biomedical Engineering, Toronto Metropolitan University, Toronto, ON M5B 2K3, Canada; hfakhrizada@torontomu.ca; 2 Department of Chemical Engineering, Toronto Metropolitan University, Toronto, ON M5B 2K3, Canada

**Keywords:** biodegradable polymers, polyolefin degradation, polylactic acid (PLA), polyhydroxyalkanoates (PHA), oxo-biodegradable plastics, pro-degradant additives, abiotic and biotic degradation, compostability standards, circular economy, sustainable plastic alternatives

## Abstract

The widespread use of single-use plastics, particularly polyethylene (PE) and polypropylene (PP), has resulted in severe environmental pollution due to their durability and resistance to degradation. This report reviews current degradable alternatives to conventional polyolefins and strategies for enhancing their breakdown in natural and managed environments. Mechanisms of abiotic and biotic degradation are examined alongside the influence of environmental factors and standardized testing protocols. Commercially available biodegradable polymers—such as polylactic acid (PLA), polyhydroxyalkanoates (PHAs), poly(butylene succinate) (PBS), poly(butylene adipate-co-terephthalate) (PBAT), starch-based plastics, cellulose derivatives, chitosan, and protein-based materials—are evaluated for their sources, degradation behavior, applications, scalability, and limitations. In addition, modification techniques for PE and PP, including copolymerization, pro-degradant additives, blending with biodegradable fillers, surface functionalization, enzyme-assisted degradation, and photocatalytic additives, are critically assessed for their potential to reduce environmental persistence. Key challenges such as performance trade-offs, incomplete degradation, ecotoxicity, cost, scalability, and end-of-life management are discussed within the context of circular economic integration. This report concludes with future research directions aimed at developing cost-effective, high-performance materials that degrade completely under real-world conditions while minimizing ecological impacts.

## 1. Introduction

Single-use plastics have become extremely common due to their convenience and low cost. However, their environmental toll is immense. Since the 1950s, over 8.3 billion metric tons of plastic has been produced, of which about 6.3 billion tons has already turned to waste; however, only around 9% of this plastic waste has been recycled, while ~79% accumulates in landfills or the environment, with the remaining 12% being incinerated [1]. Polypropylene (PP) (repeating unit: –[CH_2_–CH(CH_3_)]–_n_) [2] and polyethylene (PE) (repeating unit: –[CH_2_–CH_2_]–_n_) [3] are among the most produced plastics, together constituting a large fraction of all plastics (PE alone accounts for roughly 30% by volume [1]. Their popularity stems from their excellent material properties, as PP and PE are lightweight, strong, chemically inert, and moisture resistant. These attributes make them ideal for packaging, consumer goods, and medical uses, but they also make it difficult for nature to break them down. Conventional PP and PE are non-biodegradable polyolefins with long hydrocarbon chains that persist for decades or longer. Consequently, billions of disposable bags, bottles, and food containers made of PP/PE accumulate in our oceans and land ecosystems each year. This persistent waste harms wildlife and marine life, and over time, it fragments into microplastics that infiltrate food webs and even human drinking water. The linear “take–make–dispose” economy for plastics is clearly unsustainable [1].

In response to rising plastic pollution, there is an increasing emphasis on developing degradable and biodegradable plastic alternatives to reduce the long-term environmental contamination of plastics [4,5,6]. Biodegradable polymers are designed to degrade into carbon dioxide, water, methane, and biomass via microbial activity under suitable environmental conditions [7,8].

Oxo-degradable polyolefins are formulated with transition-metal pro-oxidant additives (1–5 wt.%) to catalyze oxidative fragmentation when exposed to UV, heat, or oxygen [6,8]. However, fragmentation does not guarantee full mineralization, and residual microplastics may persist long-term in the environment [8,9]. Additionally, circular economy strategies, particularly increasingly efficient recycling and reuse systems, are critical for curbing new plastic waste generation [1]. This review focuses on degradable single-use plastics, examining PP and PE degradation mechanisms (abiotic/biotic), environmental influences on breakdown rates and completeness, and polymer modifications including additives, copolymerization, physical surface treatment, and enzyme incorporation [6,10].

Specifically, this review will (1) explore abiotic (UV-driven photo-oxidation, thermal oxidation, hydrolysis) and biotic (microbial, enzymatic) degradation pathways across different environments [7,9,10]; (2) evaluate promising biodegradable replacements for PE/PP, including PLA, PHA (e.g., PHB), starch-based blends, and natural composites, and assess their degradation performance, lifecycle impacts, and industrial feasibility [5,7]; (3) examine structural modification approaches to enhance polyolefin degradability, such as pro-oxidant additives, copolymerization, surface functionalization, and enzyme facilitation, and quantify their influence on material properties and breakdown rates [6,8,10]; and (4) discuss the remaining challenges such as balancing material design with performance requirements, preventing microplastic residue formation, minimizing lifecycle greenhouse gas emissions, and ensuring compatibility with current recycling infrastructure [1,7]. We conclude with proposed research directions, like standardized field degradation testing, combined recycling–degradation workflows, and enzyme-catalyzed polyolefins, to accelerate the development of genuinely sustainable plastics. Given that only 9% of the 8.3 billion tons of global plastics produced since the 1950s have been recycled [1], immediate mitigation and long-term innovations in materials science are urgently required.

## 2. Mechanisms of Plastic Degradation

Plastic degradation can occur through abiotic mechanisms, biotic mechanisms, or more commonly a combination of both [10,11,12]. Abiotic degradation refers to the physicochemical breakdown of polymers without direct biological action, primarily involving photo-oxidation by sunlight, thermal oxidation (heat exposure), hydrolysis (reaction with water), and mechanical fragmentation [10,11,13]. Biotic degradation (biodegradation) involves microorganisms, typically bacteria and fungi, which secrete enzymes capable of depolymerizing plastic materials into simpler molecules [12,13,14]. These abiotic and biotic pathways often act sequentially or synergistically. For instance, biodegradation of polyolefins such as polyethylene (PE) first requires abiotic oxidative processes (primarily UV-induced) that introduce functional groups like carbonyls into the polymer structure, thus enabling microbial degradation [10,11,15]. This concept of abiotic weathering facilitating subsequent microbial biodegradation is termed two-step degradation, particularly prominent in oxo-biodegradable plastics [10,12,14].

Abiotic degradation predominantly occurs through photo-oxidation induced by ultraviolet (UV) radiation in sunlight [13,15,16]. UV photons generate free radicals in polyolefins, initiating reactions with atmospheric oxygen, forming peroxides and carbonyl groups, and ultimately causing chain scission and loss of mechanical integrity [10,15,16]. Ojeda et al. [16] demonstrated that common polyolefins (HDPE, LLDPE, PP) exposed to outdoor weathering lost nearly 90% of tensile strength within several months and virtually all mechanical integrity within a year, with PP degrading fastest due to its tertiary carbon backbone structure. Thermal oxidation is related and can occur at elevated temperatures (60–100 °C) typical in industrial processing or composting environments, similarly promoting chain scission [10,12,13]. Hydrolytic degradation mainly affects plastics containing hydrolysable functional groups (esters, amides, acetals), such as polyesters (PLA, PET), whereas pure hydrocarbons like PE and PP are resistant [11,12,14]. Mechanical fragmentation due to physical stresses (waves, abrasion) further breaks down larger plastic items into microplastics, facilitating subsequent abiotic and biotic degradation by increasing exposed surface area [10,11,14].

Biotic degradation of plastics involves microorganisms using the polymer as a carbon source and converting it into CO_2_ (under aerobic conditions) or methane (in anaerobic environments), along with biomass and water [11,12,14]. Microbes accomplish this through enzymes, such as lipases, esterases, and various depolymerases, that cleave ester bonds found in biodegradable polyesters like PLA, PCL, and PHAs [12,14,17]. Microbial enzymes such as amylases and cellulases also degrade natural polymers like starch and cellulose [11,14,17]. In contrast, standard plastics with carbon–carbon backbones (e.g., polyolefins, PVC, polystyrene) are highly hydrophobic and were traditionally considered non-biodegradable due to their resistance to enzyme interaction [11,14,18]. However, over the last two decades, studies have identified bacterial and fungal strains, such as Oscillatoria (cyanobacteria), Streptomyces, Brevibacillus, and diverse fungi, that can slowly metabolize oxidized PE and PP substrates [11,14,18]. Notably, Sanluis-Verdes et al. [19] discovered two phenol oxidase enzymes in the saliva of *Galleria mellonella* (waxworm) larvae capable of directly oxidizing and depolymerizing polyethylene within hours at an ambient temperature and neutral pH, overcoming the critical initial oxidation barrier [11,16,19,20].

In natural environments, plastic degradation is a complex, multi-stage process. Biodeterioration often begins with abiotic weathering, UV radiation, heat fluctuations, and mechanical stress, which cause visible modifications such as color fading, micro-cracks, and surface embrittlement [11,13,14]. Biotic factors, including fungal colonization and microbial biofilm formation, further alter the polymer surface by trapping moisture or altering the pH [11,14,17]. Subsequent oxidation or hydrolysis reduces molecular weight, a process often termed “degradation” in a strict sense [11,12,14]. The final biodegradation stage involves microorganisms fully mineralizing the fragments into CO_2_, methane, water, and biomass [12,14,17]. In starch-based plastics, these stages can overlap and proceed rapidly. However, for resistant polymers such as PE/PP, the primary bottleneck remains the initial oxidative breakdown, driving current research on enzymatic catalysts and additive-based priming [14,18,19]. Degradation rates are highly environment-dependent, and plastics that degrade in controlled lab settings within weeks may persist for decades in cooler, darker marine conditions [11,12,14]. Therefore, a mechanistic understanding of these pathways across varying environmental contexts is essential for accurately predicting plastics’ fate.

## 3. Environmental Conditions for Degradation and Standards

The rate and extent of plastic degradation depend strongly on environmental conditions such as temperature, UV exposure, oxygen availability, moisture, microbial presence, and exposure duration [11,14,21]. Industrial or managed environments, like composting facilities, offer high temperatures (~58 °C), high humidity, and abundant microbial activity in oxygen-rich settings, which accelerate breakdown [14,21,22]. In contrast, natural or home settings (open land, soil, marine water) feature lower and more variable temperatures, moisture levels, and microbial populations, generally showing slower degradation [11,14,21]. Standardized laboratory tests, such as ASTM D5338 [23], ASTM D6400 [24], ISO 17088 [25], EN 13432 [26], ASTM D5988 [27], and AS 5810 [28], simulate these environments to assess biodegradability under controlled conditions [21].

In industrial composting, materials like PLA or starch-based bioplastics typically achieve >90% biodegradation to CO_2_ within a few months at ~58 °C, as required by ASTM D6400 and ISO 17088 standards [21,22]. By contrast, home composting occurs at lower ambient temperatures (~20–30 °C), with less optimized moisture and microbial communities, meaning PLA often fails to fully or rapidly degrade [14,21]. Studies show PLA remains largely intact after 12 weeks in soil at ~23 °C, whereas PHA blends display earlier signs of biodegradation under similar conditions [11,14,21]. To address this disparity, standards like ASTM D5988 (soil) and home compost norms such as AS 5810 and OK Compost HOME set benchmarks for 90% CO_2_ conversion within 12 months at ~20–30 °C [21].

## 4. Soil and Landfill Conditions

In natural soil, temperatures are ambient, and oxygen and moisture may be limited, especially a few centimeters below the surface. Soil microbial communities are diverse but less concentrated than in compost. ISO 17556 [29] is a standard test for aerobic biodegradation in soil, typically conducted at 20–30 °C by measuring CO_2_ evolution over 6+ months [30,31,32]. Polyesters like PBAT (poly(butylene adipate-co-terephthalate)) and PHA (polyhydroxyalkanoates) have shown substantial biodegradation in soil tests, whereas PLA degrades much more slowly in soil (years) unless the soil is very warm [29,30,31,32]. In landfills, conditions are largely anaerobic (especially in modern sanitary landfills). Conventional plastics may persist for decades in landfills due to the lack of oxygen and lower microbial activity; even “compostable” plastics like PLA or starch materials may barely break down anaerobically, or they generate methane when they do [14,32,33]. ASTM D5511 [34] is a test method for anaerobic biodegradation (high-solid anaerobic digestion conditions), measuring biogas production [33]. Notably, most biodegradable plastics are not designed for landfill conditions—a point of criticism since landfilling is common. Some polyesters (PHA) can anaerobically biodegrade, but others (PLA) may not [14,32,33]. Thus, a plastic that is certified compostable might still be inert in a landfill. Increasingly, researchers emphasize that proper waste management is needed to ensure biodegradable plastics actually see conditions where they can degrade (e.g., sent to compost instead of landfill) [14,32,33].

## 5. Marine and Aquatic Environments

The marine environment is particularly harsh for plastic degradation. Sunlight penetration and temperatures in the ocean are low, especially below the surface, and nutrient levels can be low, limiting microbial action [33,35,36]. A “biodegradable” plastic on land might persist for many years in seawater. For instance, poly(ɛ-caprolactone) (PCL) and polyhydroxyalkanoates (PHAs) are among the few plastics known to biodegrade in marine conditions on a timescale of months to a couple of years, whereas PLA shows essentially no biodegradation in seawater over year-long tests [35]. ASTM D6691 [37] is a standard test for aerobic biodegradation of plastics in seawater (usually performed at 30 °C in the lab), and more recently, ASTM D7991 [38] covers marine sediment environments [31,33]. Dilkes-Hoffman et al. [35] reported that even supposedly biodegradable plastics have highly varying decay rates in the ocean: PHA films could biodegrade ~90% in under a year in warm marine waters, while PLA samples showed <1% biodegradation in the same period [35]. Consequently, a new generation of polymers (and test standards) focuses on marine biodegradability—for example, the ISO 22403:2020 [39] specifies requirements for biodegradable plastics in marine environments [31,33]. European and global regulatory bodies are cautious: the EU Single-Use Plastics Directive (2019) notes that claiming biodegradability is not a free pass unless the material truly biodegrades in natural settings like oceans, to avoid giving a false sense of security about littering [33,35].

## 6. Standardized Test Methods

To ensure uniform evaluation of biodegradability, several ASTM and ISO methods are used by researchers and industry. A few key ones include ASTM D6400 (industrial compostability certification, as mentioned), ASTM D5338 (determines aerobic biodegradation of plastics under controlled composting conditions, very similar to D6400), ASTM D5988 (CO_2_ evolution in soil) [30], ASTM D6691 (marine biodegradation in seawater, laboratory) [35], and ISO 14855 [40] (another aerobic compost biodegradation test). These tests typically involve measuring the carbon conversion to CO_2_ over time (for aerobic tests) or methane (for anaerobic). Passing criteria are often ≥60–90% conversion within a specified time compared to a natural reference like cellulose. Another important standard is ASTM D6954 [41], a multi-tiered test guideline for oxo-degradable plastics. ASTM D6954 outlines a series of steps: (1) abiotic oxidation in a lab (e.g., expose sample to UV or heat until significant oxidative embrittlement), (2) followed by biodegradation testing of the oxidized fragments in soil or compost (measuring CO_2_ evolution), and (3) ecotoxicity tests on the resulting residues. This comprehensive approach is used to validate oxo-biodegradable claims—the material must not only fragment but also show microbial consumption of the fragments. For example, an oxo-PE film with metal additives would be subjected to accelerated weathering until its carbonyl index (a measure of oxidation by FTIR) reaches a certain level, then buried in soil and monitored for CO_2_ over 6–12 months. Such protocols ensure that “oxo” plastics are tested beyond just disintegration.

It must be emphasized that passing a lab test does not always guarantee a similar performance in nature. Transferability to real conditions is often poor. Haider et al. [42] pointed out that many biodegradation tests use optimal or “accelerated” conditions that may overestimate rates seen in the field. There is a push for more environmentally authentic field testing, such as deploying plastic samples in real compost piles, soils, or ocean environments and tracking their degradation (physically and chemically) over time. These studies, while slower and logistically harder, can reveal unanticipated factors (e.g., biofilm formation inhibiting degradation, or seasonal effects). For instance, field trials of “biodegradable” plastic mulch films in agriculture found that fragments can remain in soil for multiple growing seasons if conditions (cold winters, dry periods) halt microbial activity, even though the product met laboratory composting specifications [30]. Thus, understanding the required conditions for complete degradation is critical. Generally, a truly biodegradable plastic should be matched to an appropriate disposal route: if a material only degrades in industrial compost, it needs to actually be collected and composted; if it is marketed for open-environment use (like mulch films, fishing gear), it should be proven to biodegrade in those environments to avoid persistent litter. Standardizing such environment-specific labels (e.g., “soil biodegradable”, “marine biodegradable”) is an ongoing effort in the international community [32].

## 7. Biodegradable Alternatives to PP and PE

**Figure 1:**
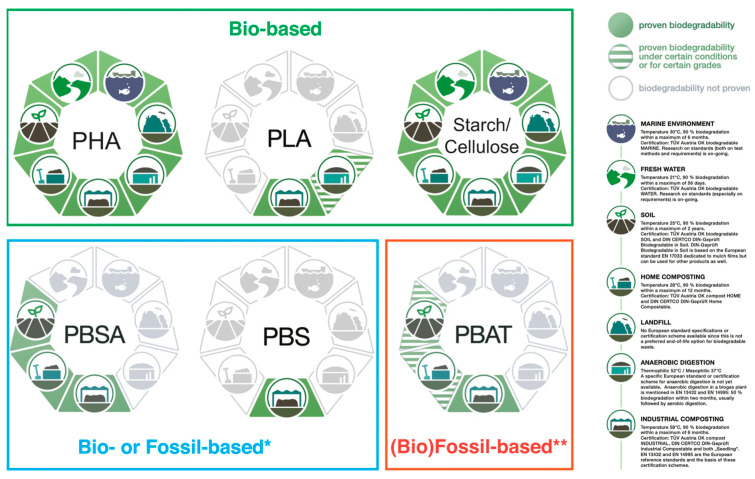
Biodegradability of bio-based and fossil-based biodegradable polymers in various environments. The schematic compares polymers such as PHA, PLA, starch/cellulose, PBSA, PBS, and PBAT, indicating proven biodegradability (green), partial biodegradability (light green), and environments where biodegradation has not been demonstrated (gray). Environments include marine, freshwater, soil, home composting, landfill, anaerobic digestion, and industrial composting. * Polymers can be 100% biobased, 100% fossil-based, or a blend of bio-based and fossil-based; ** Polymer components can be 100% bio-based or 100% fossil-based [45]. Licensed under CC BY 4.0.

A variety of biodegradable polymers have been developed as alternatives to conventional polyolefins, encompassing both bio-based materials (derived from renewable biomass) and petrochemical-derived biodegradable polymers [21,43,44] (Figure 1). These include aliphatic polyesters (such as polylactic acid and polybutylene succinate), microbially produced polyesters (PHAs), starch-based plastics, and naturally derived polymers like cellulose, chitosan, and protein-based plastics [21,43,44]. For each, we consider their sources, degradation behavior, production scale, and challenges like cost and performance [21,43].

Polylactic Acid (PLA):
Repeating unit: –[C_3_H_4_O_2_]–_n_, structure: –[–O–CH(CH_3_)–CO–]–_n_

**Figure 2:**
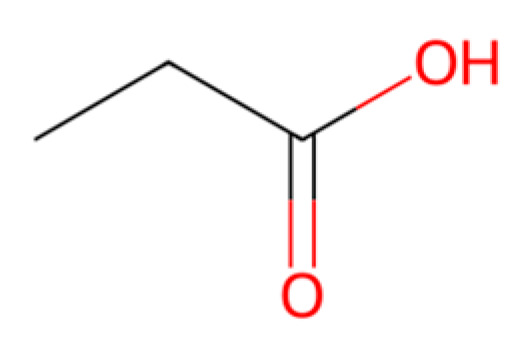
PLA 2D chemical structure [3].

PLA is one of the most widely used biodegradable plastics today, often found in packaging, disposable cutlery, and compostable bags. It is a thermoplastic aliphatic polyester made by fermenting sugars (usually from corn or sugarcane) to lactic acid, then polymerizing lactide monomers [21,43]. PLA is bio-based (from renewable feedstock) and biodegradable under appropriate conditions. In industrial composting (~58 °C, high humidity), PLA items can disintegrate and achieve >90% biodegradation within 2–3 months [3,21], but under lower-temperature or marine conditions, PLA’s degradation is much slower [21]. Its breakdown proceeds via hydrolysis of the ester backbone, which is visible in Figure 2, into lactic acid monomers, which are then metabolized by microbes [21,43]. Lifecycle and scale: The production of PLA has scaled rapidly in recent years—global PLA manufacturing capacity was about 0.46 million tons in 2022 and is projected to reach ~0.79 million tons by 2027 [21,46]. This is still a small fraction of plastics (for comparison, PP/PE are produced in the tens of millions of tons each annually), but PLA’s market is growing ~18–20% annually [46]. PLA is relatively costly, currently around US USD 2000 per ton, several times higher than commodity PE/PP resins, due to the fermentative production process and polymerization costs. Its properties (high strength, glossy clarity, processability) are comparable to PET or polystyrene, making it a good candidate for certain single-use items [43,47]. A drawback is PLA’s brittleness and low heat resistance (T_g ~ 60 °C), which limit some uses. New “toughened” PLA blends and PLA copolymers are being developed to broaden its application range (e.g., PLLA-PDLA stereocomplex PLA can have higher heat resistance). From a sustainability view, PLA can reduce fossil resource use and net CO_2_ emissions, especially if the feedstock is agricultural waste and if composting is available for disposal. If PLA goes to landfill, it might not degrade and could even contribute to methane emissions if anaerobically degraded; thus, proper composting infrastructure is important for its environmental benefits to be realized [3,21,43].

Polyhydroxyalkanoates (PHAs):
General structure: –[O–CHR–CH_2_–CO]–_n_, e.g., PHB: –[O–CH(CH_3_)–CH_2_–CO]–_n_ [48]

**Figure 3:**
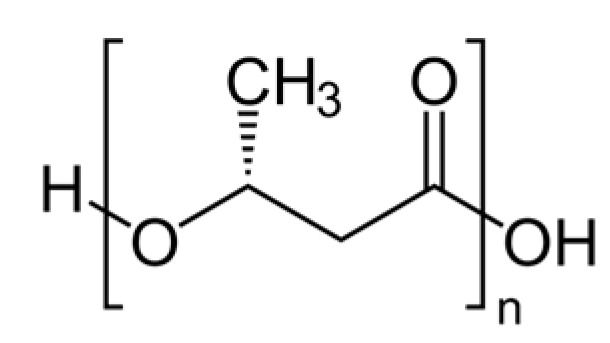
PHA 2D chemical structure [48].

PHAs are a family of biopolyesters produced naturally by various bacteria as energy storage compounds. Examples include poly(3-hydroxybutyrate) (PHB) and its copolymers like PHBV. PHAs are bio-based, biodegradable, and even biocompatible, and they can biodegrade in a wide range of environments (soil, compost, and even marine) given the right microbes. Its chemical structure can be seen above in Figure 3. PHB was one of the earliest bioplastics discovered (commercialized in the 1980s by ICI as “Biopol”). It has properties somewhat similar to polypropylene (semi-crystalline, but more brittle). Newer copolymer PHAs (PHBV, PHBHHx, etc.) have improved flexibility and toughness. PHAs are readily consumed by soil bacteria and marine microorganisms—for instance, PHBV mulch films left in soil will typically biodegrade substantially over a growing season, eliminating the need to retrieve them. In marine tests, PHAs can achieve significant biodegradation within a year whereas PLA does not, making PHAs attractive for applications where plastics might end up in nature (e.g., fishing gear, agricultural films). The challenge for PHAs is cost and scalability: they are produced via microbial fermentation of sugars or oils, followed by extraction from the cells. This process is currently expensive (PHAs can cost USD 4000–8000 per ton), and global production is still under ~0.05 million tons/year, though it is expected to rise as new facilities come online. Companies are exploring using methane or waste oils as feedstocks for cheaper PHA production. In terms of their life cycle, PHAs are biodegradable even in anaerobic digesters (yielding biogas), offering a potential end-of-life valorization. They are also non-toxic and even used in medical devices (sutures, patches) that safely resorb in the body. Continued R&D aims to improve the yield and reduce downstream processing costs for PHAs, as well as to tailor their properties (through co-monomer composition) for different uses.

Poly (butylene succinate) (PBS) and other Biodegradable Polyesters:
Repeating unit: –[O–(CH_2_)_4_–O–CO–(CH_2_)_2_–CO]–_n_ [49]

**Figure 4:**
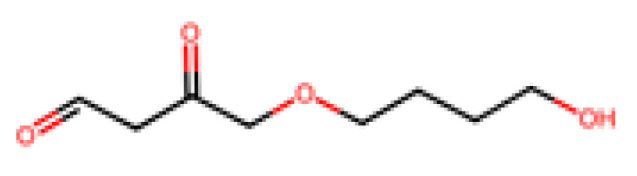
PBS 2D chemical structure [49].

**Figure 5:**
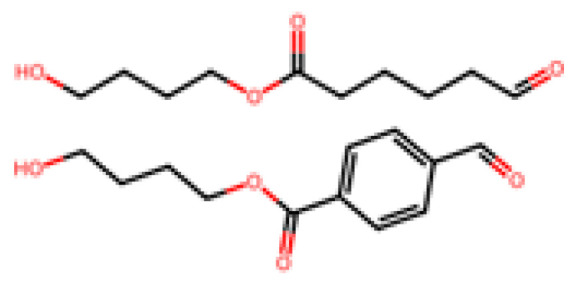
PBAT 2D chemical structure [50].

PBS, illustrated above in Figure 4, is an aliphatic polyester (often bio-based now, from bio-succinic acid) that is biodegradable and used in packaging and agricultural films. It has good thermal and mechanical properties (more ductile than PLA) and biodegrades in compost and soil, though is perhaps slower than PLA. Another related polymer is PBAT, illustrated in Figure 5.

**Figure 6:**
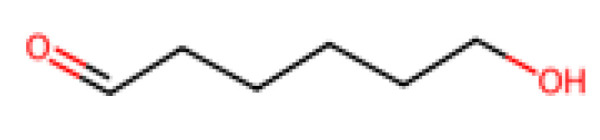
PCL 2D chemical structure [51].

Poly(butylene adipate-co-terephthalate) is a copolymer of butylene adipate and butylene terephthalate—which is actually partly petrochemical (it contains adipate and terephthalate units) but is engineered to be biodegradable. PBAT is very flexible (used in compostable bags) and degrades fairly well in soil and compost (it can pass ASTM D6400). Its biodegradation in marine life is limited but somewhat better than PLA. PBAT is often blended with PLA or starch to improve flexibility. PCL, shown below in Figure 6 (polycaprolactone) (repeating unit: –[O–(CH_2_)_5_–CO]–_n_) [51], is a fossil-derived polyester that is fully biodegradable (even at ambient temperature, though slowly).

PCL has a very low melting point (~60 °C) and is extremely flexible; thus, it is used as a compatibilizer or in niche medical applications. PCL will biodegrade in compost or soil given enough time and is readily attacked by lipase enzymes. However, due to its cost and low strength, it is not widely used alone in bulk applications. Polyethylene succinate (PESu) (repeating unit: –[O–(CH_2_)_2_–O–CO–(CH_2_)_4_–CO]–_n_) [49] and poly(butylene succinate-co-butylene adipate) (PBSA), copolymers with succinate and adipate units, are other variants with biodegradability. Collectively, these synthetic biodegradable polyesters often share a common trait: they break down by microbial enzymatic hydrolysis of their ester bonds, ultimately metabolized to CO_2_. They tend to require less rigorous conditions than PLA for biodegradation (some degrade at lower temp. or in soil), but exact rates vary. Many are commercially sold as blends—e.g., BASF’s Ecoflex is PBAT, usually blended with PLA to make Ecovio, and Novamont’s Mater-Bi is a starch-PBAT blend. Production volumes of PBAT and PBS are in the order of a few hundred thousand tons globally and rising.

Starch-Based Plastics:
Polymer of α-D-glucose units linked mainly by α(1 → 4) bonds

**Figure 7:**
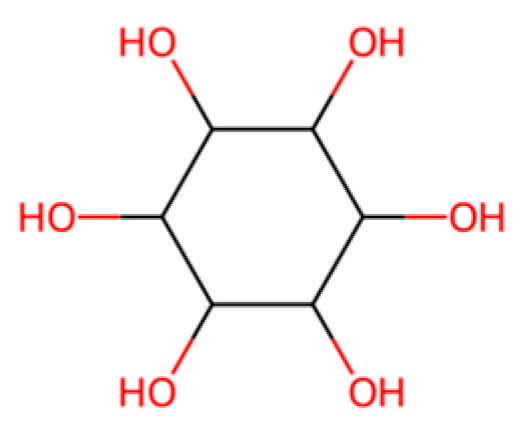
Starch 2D chemical structure [54].

Starch, shown in Figure 7 above, a natural polymer from corn, potato, etc., has long been used in biodegradable materials. Thermoplastic starch (TPS), made by plasticizing starch with glycerol or other agents, can be formed into films and products [7]. On its own, TPS is highly biodegradable; the starch is readily consumed by microbes, often ~90% degraded to CO_2_ in a few weeks in soil/compost [7]. However, pure starch plastics are brittle and sensitive to water [7]. Therefore, starch is often combined with other polymers. One common approach is blending starch with polyester (like PBAT or PLA); the starch reduces cost and increases biodegradability, while the polyester provides strength and water resistance [7,52,53]. For instance, some compostable bag formulations contain 40–50% starch with PBAT and PLA [7]. These blends can be processed on conventional plastic equipment and will compost in municipal facilities [7]. The presence of starch can also slightly “seed” biodegradation: microorganisms attack the starch granules first, leaving a porous matrix that allows better access to the synthetic polymer portion. Another application is starch foam packing peanuts, which dissolve in water and biodegrade (these are essentially extruded starch with minor additives) [7]. From a lifecycle perspective, starch-based plastics use renewable feedstock and can have low greenhouse footprints if land use is managed, but they may compete with food resources if based on edible crops [7]. Research is ongoing into using agricultural waste starch or cellulose (e.g., cassava peels, bagasse) to avoid competing with food [7]. Starch-based materials generally cannot match the durability of PE/PP (they have poor moisture resistance), so their use is focused on short-life, disposable items where biodegradability is prioritized over longevity [7].

Cellulose and Derivatives:
Polymer of β-D-glucose units linked by β(1 → 4) bonds [55]

**Figure 8:**
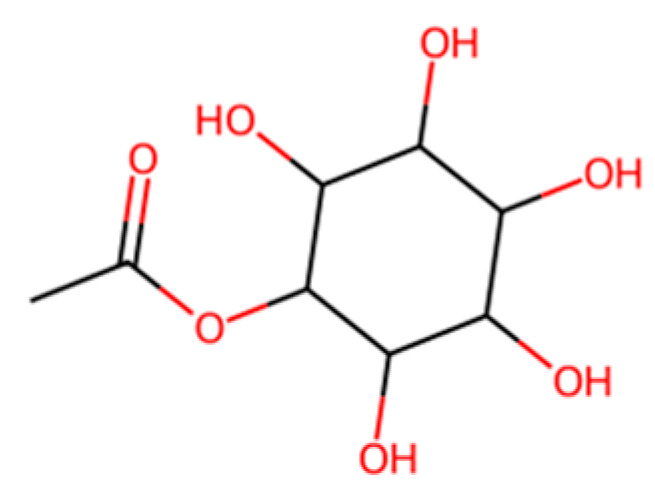
Cellulose acetate 2D chemical structure [55].

Cellulose, the abundant polysaccharide from plants, is naturally biodegradable (compostable, soil-degradable) because many microbes produce cellulase enzymes [56]. Unmodified cellulose (like paper) degrades relatively quickly. In the plastics context, cellophane, a regenerated cellulose film, is a classic “bio-origin” plastic that is biodegradable [56]. Cellophane was used for packaging before petroleum plastics but fell out of favor due to cost and performance issues (stiffness, tearing when dry) [56]. It is seeing a minor revival for specialty sustainable packaging. Cellulose acetate (acetylated cellulose with varying degree of substitution), a derivative used in films and fibers, can be biodegradable or not depending on the degree of substitution, and highly acetylated cellulose is slower to biodegrade [56]. Cigarette filters made of cellulose acetate, shown in Figure 8, biodegrade only over years because of their acetylation and plasticizer content [56]. Reducing acetylation yields a material that soil microbes can break down more readily [56]. Some companies offer “biodegradable” cellulose acetate straws or eyeglass frames with lower substitution levels. Another interesting material is Rayon, which is effectively pure cellulose in fiber form; it biodegrades in soil/marine environments similarly to cotton [56,57]. Generally, cellulose-based plastics are bio-based and truly biodegradable, but they often have inferior mechanical properties or a higher cost compared to polyolefins [56]. They may also be sensitive to water unless chemically modified [56]. New research is exploring cellulose nanofibers and composites to improve strength, or hybrid materials like cellulose–polyester blends.

**Figure 9:**
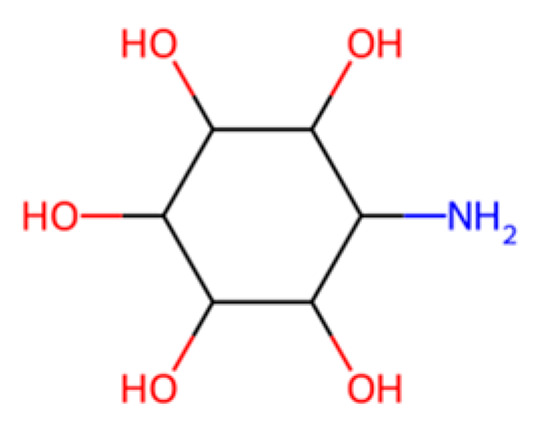
Chitosan 2D chemical structure [58].

Chitosan, shown above in Figure 9 (polymer of β-(1 → 4)-linked D-glucosamine [58] (partially deacetylated chitin)), derived from chitin via deacetylation, is a poly-D-glucosamine polysaccharide that is biodegradable and exhibits inherent antimicrobial activity [18,59]. Chitosan films have been extensively studied as edible coatings and active packaging materials capable of extending food shelf life [18,60]. Despite good film-forming ability, they are water-soluble under acidic conditions and generally lack the mechanical robustness of synthetic plastics, limiting their use to niche rather than bulk packaging applications [60]. Blending chitosan with other biodegradable polymers (e.g., starch, proteins) or reinforcing agents has been shown to improve functional properties of packaging films [61].

**Figure 10:**
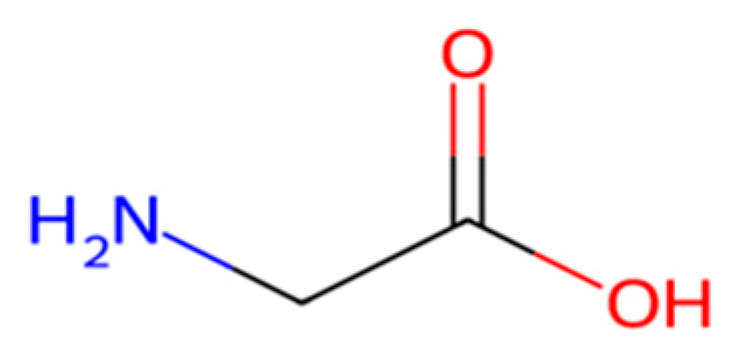
Generic amino acid 2D chemical structure [66].

Protein-based plastics made from gelatin, casein, or soy protein are inherently biodegradable polypeptides but typically suffer from poor mechanical strength and high moisture sensitivity [62]. Historically, casein was used on an industrial scale in the early 1900s to manufacture hard, molded items such as buttons and ornaments. Modern whey protein and casein films are researched for food packaging because they offer excellent oxygen barrier properties under low-humidity conditions, though they require significant plasticizers or crosslinking to improve water resistance additives, which can compromise biodegradability or safety [63,64,65] (Figure 10).

**Figure 11:**
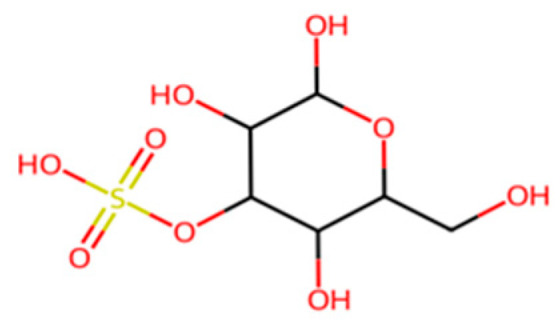
Agarose 2D chemical structure [67].

**Figure 12:**
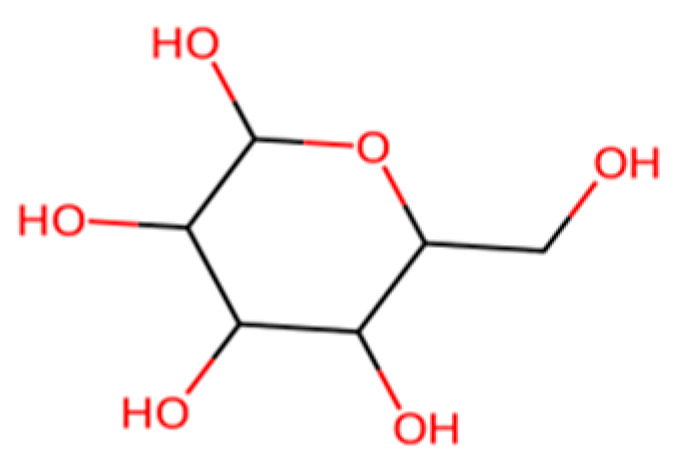
Carrageenan 2D chemical structure [68].

**Figure 13:**
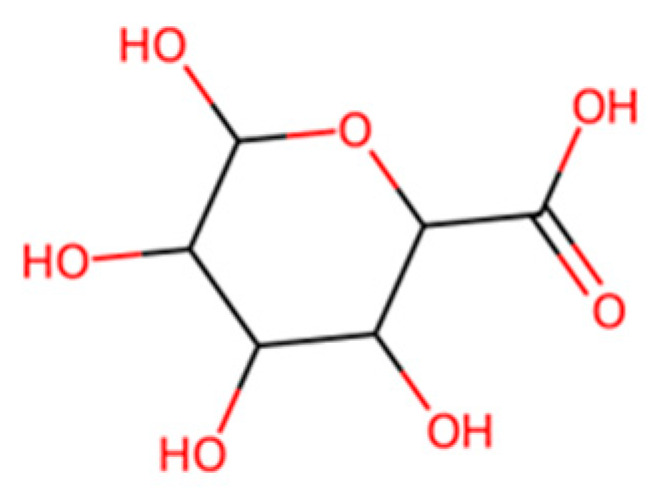
Alginate 2D chemical structure [69].

Algal polysaccharides such as agar, alginate, and carrageenan, shown in Figure 11, Figure 12 and Figure 13 below, can form biodegradable films and are being explored for edible packaging applications, like seaweed-based water capsules, that maintain food quality and extend shelf life. While these biopolymers are environmentally benign, their mechanical performance and moisture resistance lag far behind polyolefins, so they are often envisioned as multi-layer barrier layers or components in composite systems [64] (Table 1 and Table 2).

In summary, there is no one-size-fits-all replacement for PE and PP yet. Each biodegradable alternative has pros and cons. Polyesters like PLA and PBAT mimic many properties of conventional plastics and fit existing processing but demand specific end-of-life handling (composting). PHAs offer better environmental degradability but are expensive and still being optimized for large-scale use. Starch and natural polymers are eco-friendly and cheap, but struggle in performance. Ongoing research and industrial innovation aim to improve material properties, reduce costs (e.g., cheaper feedstocks, new catalysts or microbes), and ensure that these alternatives truly degrade in real disposal scenarios. Importantly, any switch to biodegradable plastics should be accompanied by proper waste management—for instance, clearly labeling and routing compostables to compost facilities—to avoid contaminating recycling streams or leaving them in environments where they do not break down. In parallel, improving the recyclability of existing polymers and designing products for longer use are key strategies. Next, we turn to how the polymers PP and PE themselves might be modified to be more degradable, since completely phasing them out may not be immediately feasible in all applications.

## 8. Structural Modification of PE and PP for Enhanced Biodegradability

Conventional polyethylene and polypropylene are chemically inert and resistant to microbial attack, primarily because of their simple carbon–carbon bond backbone and hydrophobic, high-molecular-weight nature [11,81]. A major area of research is exploring ways to alter the structure or formulation of PE/PP so that they become more susceptible to degradation. Several strategies have emerged: copolymerization to insert degradable linkages into the polymer chain, blending with additives (such as pro-degradant additives or biodegradable fillers) to induce or accelerate breakdown, surface or chemical treatments to introduce functional groups, and even embedding catalysts or enzymes to trigger degradation [10]. In this section, we discuss these approaches and illustrate each with recent examples from the literature.

## 9. Copolymerization and Cleavable Bonds in the Backbone

One direct way to make polyolefins degradable is to modify their backbone to include bonds that can be cleaved by hydrolysis or other mild chemical processes. Traditional PE is –(CH_2_–CH_2_)ₙ–, an extremely stable chain. Researchers have synthesized polyolefin copolymers that contain periodic ester or ketone groups along the chain, which serve as weak links [82,83]. For example, starting in the 1980s, ethylene was copolymerized with a small percentage of carbon monoxide (CO) to yield an ethylene–carbon monoxide copolymer (often called polyketone). The carbonyl groups in that polymer undergo Norrish photochemical reactions under UV light, causing chain scission [82]. Bacteria can also more easily attack a polymer containing carbonyls [56]. Similarly, ethylene has been copolymerized with vinyl ketones or acrylate monomers to introduce degradable sites. A recent advancement reported by Zeng et al. [84] uses cyclic ketene acetal (CKA) comonomers (such as 2-methylene-1,3-dioxepane) in radical copolymerization with ethylene. This reaction inserts ester linkages directly into a predominantly polyethylene chain. By adjusting the feed ratio and conditions, they obtained a material that is mostly ethylene units but with a controlled fraction of ester-containing repeat units. The resulting copolymer behaves much like PE, but in the presence of a base or certain enzymes, the ester bonds cleave, and the polymer degrades into shorter segments [83]. Zeng et al. [84] demonstrated that their modified PE copolymer could be fully dissolved or broken down by mild treatment (e.g., with triethylamine base) into low-molecular-weight fragments. While base-catalyzed degradation is not the same as biodegradation, these cleavable PE analogues are a proof-of-concept that we can make polyolefins inherently degradable. Another example is polypropylene with hydrolysable links: there has been work on copolymerizing propylene with small amounts of polar monomers like maleic anhydride or vinyl acetate to introduce ester links, making PP more oxidatively and hydrolytically sensitive [82,83]. Overall, copolymerization must balance maintaining the desirable properties of PE/PP with adding just enough “weak links” to allow degradation. Many such modified polyolefins are still at the lab scale, but they illustrate a viable path: building in a self-destruct button at the chemical level [84].

## 10. Pro-Degradant Additives and Oxo-Biodegradable Plastics

Perhaps the most common approach attempted in commercial products is using pro-degradant additives (PDAs) in the polymer. These are typically metal compounds (often transition metal salts like cobalt stearate, manganese stearate, etc.) that catalyze the oxidation of the polyolefin when it is exposed to heat and/or UV light [56,85]. Plastics incorporating such additives are marketed as “oxo-degradable” or “oxo-biodegradable” plastics. The mechanism: the metal salts speed up the formation of free radicals in the polymer (for example, by decomposing hydroperoxides that form upon UV exposure into more radicals) [86]. This greatly accelerates the abiotic oxidative degradation phase. A polyethylene film that would normally take years to embrittle might do so in a few months of outdoor exposure if it contains a PDA [86]. The plastic then fragments into small pieces enriched with oxygen-containing groups (like ketones, carboxylic acids). The second step is that these oxidized fragments are supposedly biodegradable—microbes can attack the residual material, consuming it over time [87]. Metal-catalyzed oxidative PP/PE has been shown to reach very high carbonyl levels and lose molecular weight dramatically after weathering [56]. For example, in one study, oxo-additive HDPE film exposed to natural sunlight for ~6 months became so brittle that it disintegrated under slight stress, and when buried in soil, the pieces showed substantially higher CO_2_ evolution compared to non-oxidized PE (indicating microbial mineralization) [86]. Shah et al. [88] reviewed the role of different transition metals in catalyzing polyolefin oxidation, noting that certain metals like Co(II) and Mn(III) can be especially effective at promoting peroxidation of the polymer chains (via Fenton-like reactions). Oxo-biodegradable plastic producers typically add ~1% of a proprietary metal additive masterbatch to the resin [89].

The first stage of oxo-biodegradable plastic degradation is fragmentation, driven primarily by metal-catalyzed oxidative chain scission. Transition metal stearates (e.g., Co, Mn, Fe salts) promote free-radical formation and accelerate carbonyl group accumulation, leading to embrittlement and loss of mechanical integrity. This abiotic phase typically proceeds under UV or thermal exposure and results in a drastic reduction in molecular weight (often to <5000 Da within months under accelerated weathering) [56,86]. The polymer disintegrates into micro- and nanoscale oxidized fragments containing ketones, alcohols, and carboxylic acids. However, this stage only represents physical and chemical breakdown, not complete biodegradation. Kinetic studies generally report fragmentation half-lives of weeks to months in open-air conditions, but the rate is highly dependent on UV intensity, temperature, and the specific pro-oxidant formulation [87].

**Figure 14:**
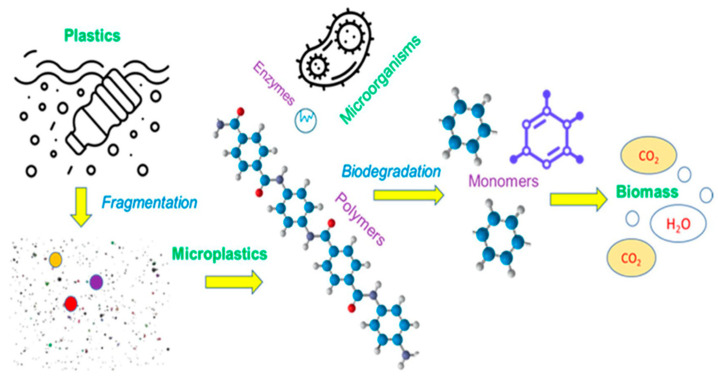
Schematic of plastic biodegradation showing fragmentation into microplastics, enzymatic depolymerization into monomers, and conversion into biomass, CO_2_, and H_2_O by microorganisms [90]. Licensed under CC BY 4.0.

The subsequent stage, mineralization, refers to the microbial conversion of oxidized residues into CO_2_, water, and biomass. This process is much slower and often incomplete. Reported CO_2_ evolution for oxo-additive polyolefins under controlled composting or soil burial rarely exceeds 1–5% of theoretical carbon conversion after 90 days, compared to >90% for certified compostable bioplastics [86]. True mineralization typically requires prior oxidation to generate low-molecular-weight species accessible to microbial enzymes. Consequently, most standards bodies (ASTM D6954, ISO 17556, EN 13432) classify oxo-degradable plastics as not fully biodegradable, and their environmental claims have been restricted or banned in several jurisdictions, including the EU and Canada, due to concerns over persistent microplastic formation (Figure 14).

## 11. Controversy and Performance

Oxo-degradable plastics have stirred debate. Critics argue that while they do fragment faster, the ultimate biodegradation is often incomplete or unproven, leading to microplastic pollution [6]. Indeed, if the fragments do not fully mineralize, one could be making the pollution problem worse by creating microplastics more quickly [56]. Supporters (and some studies) show evidence of substantial biodegradation of oxidized fragments over extended periods. For instance, Chiellini et al. [91] found that oxo-degradable plastic films lost ~70% of their mass after a couple of years in soil and appeared to be assimilated by microorganisms (supported by microbial counts and CO_2_ data). A 2005 study by Koutný et al. [89] showed that PP films with metal additives, after UV aging, not only fragmented but also that soil fungi were able to grow on and metabolize a portion of the material over months (measured by evolved CO_2_ and characteristic chemical changes) [89]. However, complete mineralization may take many years [6]. Because of these uncertainties, the EU in 2019 moved to ban oxo-degradable plastics that are marketed as biodegradable, on the grounds that they primarily contribute to microplastics [6]. Standards like ASTM D6954 were developed to precisely require proof of biodegradation, not just fragmentation [89]. Newer additive systems are being tweaked to improve biodegradability of the residues—for example, combining metal catalysts with biologically labile materials (like starch or PHA) so that microbes are attracted and co-metabolize the polymer [89]. One emerging class are additives termed “biotransformation additives”, which are claimed to convert polyolefins into a waxy, bio-assimilable substance (through some proprietary oxidation chemistry) [6]. Bagheri et al. [92] highlight that future “oxo-biodegradable” technologies need to demonstrate the complete breakdown and safety of breakdown products (no toxic metal accumulation, etc.). They also note that current developments include photosensitizing additives that respond to specific light wavelengths to ensure even buried or semi-exposed plastics can start degrading [69]. In summary, PDAs remain a practical short-term method to reduce persistence of littered PE/PP by accelerating its breakdown [86]. When properly formulated, they can substantially shorten the physical presence of plastic in the environment (from decades to a few years or less) [87]. But ensuring the fragments truly biodegrade requires careful additive choice and post-oxidation monitoring [89]. Additional challenges include the shelf life of the product (additives should not trigger degradation too early) and the impact on recycling streams (oxo-additives can contaminate recyclate and weaken it) [6]. Thus, while oxo-additives can be part of the solution, they must be used with transparency and backed by data on end-to-end biodegradation [93].

## 12. Blending with Biodegradable Polymers or Fillers

**Figure 15:**
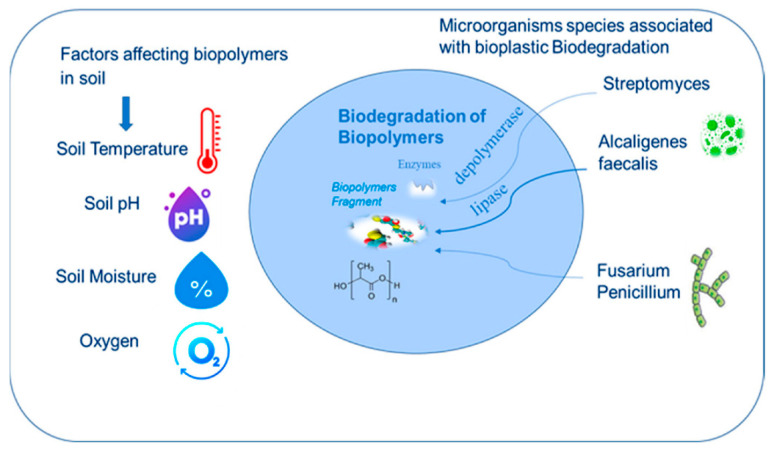
Factors influencing biopolymer biodegradation in soil. Key parameters such as soil temperature, pH, moisture, and oxygen availability affect microbial activity and enzymatic degradation of bioplastics. Enzymes like depolymerase and lipase produced by microorganisms including Streptomyces, Alcaligenes faecalis, Fusarium, and Penicillium contribute to polymer breakdown [90]. Licensed under CC BY 4.0.

Another strategy to modify PP/PE is to blend them with other materials that are more degradable. The idea is that the degradable component will either create pathways for the polyolefin to break up or at least reduce its overall environmental footprint by leaving behind less persistent material. A classic example is blending starch into PE. In fact, early “biodegradable plastic” attempts in the 1990s were often PE with 5–20% starch filler. The starch dispersed in the PE matrix can be consumed by microbes in soil, leaving behind a porous, foam-like PE structure that is more accessible to oxygen and microbes. While this alone does not make the PE biodegrade fully, it does hasten disintegration [94,95]. Modern versions of this concept include Mater-Bi (which contains some polycaprolactone or PBAT in addition to starch and a small fraction of PE in older grades). Blends of PE or PP with truly biodegradable polyesters have also been explored in research. For instance, blending PP with PLA or PCL: the PLA domains can hydrolyze and biodegrade, theoretically helping to break the PP phase into smaller chunks. One 2018 study showed that a PP-PHBV blend, when buried in soil, saw the PHBV fraction biodegrade within a year, and the PP portion showed some evidence of surface erosion (perhaps aided by the presence of degrading PHBV) [96]. However, complete biodegradation of the PP component was not achieved, the blend simply resulted in a microporous PP residue after the biopolymer was gone. Similarly, PE-PHA blends have been tested. The challenge is that most polyolefin blends are immiscible—they form separate phases, so the interaction is mainly physical, not chemical. If one phase goes away, it does not automatically make the other phase biodegradable, although it increases surface area [97] (Figure 15).

Another approach is using biodegradable fillers or fibers. For example, compounding PE with wood flour or cellulose fibers: the organic filler can degrade, leaving the plastic matrix more exposed. Likewise, blending PE with algae biomass or protein waste (e.g., soy hulls) has been tried. These composites can have enhanced susceptibility to biodegradation relative to neat PE [95,98]. There have also been attempts to crosslink polyolefins with biodegradable segments. For instance, grafting PP with gelatin and then crosslinking into a network—the gelatin segments might enzymatically degrade, breaking the material apart. But such complex modifications often compromise the material’s mechanical properties before yielding a substantial biodegradability benefit [14].

In practice, the most successful use of blending is in multi-component systems where the polyolefin portion is small. For example, some compostable films contain a minority of PE just to improve moisture resistance or strength, with the bulk being PBAT and starch, which biodegrade. After composting, a tiny amount of PE (~5% or less) might remain as micro-fragments that are hopefully consumed over a longer time (or filtered out in compost refining). If the PE fraction is low, the product can still meet compostability criteria because 90%+ of carbon is converted to CO_2_ from the other components. However, this is not a true solution for the polyolefin itself; it is more of a dilution strategy.

One exciting area is reactive blending, where during processing, a compatibilizer causes some bonding between PE (or PP) and the biodegradable polymer: for instance, using a graft polymer that links the phases, potentially creating block or graft copolymers in situ. These compatibilized blends may allow the biodegradable segments to “carry” attached PE chains into microbial metabolism. Research by Boughrara et al. [99] on PE-PCL reactive blends showed that after PCL biodegraded, the PE fragments were much smaller and some oxidation of PE occurred concurrently, suggesting a slight enhancement of PE biodegradability [98]. Still, the consensus is that blending alone cannot fully solve polyolefin persistence unless the blend includes other measures (like pro-oxidants or special reactive groups). Yet, blends are attractive because they are immediately applicable using existing polymers—processors can mix a percentage of bioplastic into PE or PP to make a product with somewhat reduced environmental impact. As the cost of bioplastics drops, higher blend ratios could be used.

## 13. Surface Functionalization and Chemical Treatments

Because much of polyolefins’ resistance to biodegradation comes from their hydrophobic, inert surface, altering just the surface chemistry can increase interactions with microbes or water [100,101]. Surface functionalization techniques include plasma treatment, ozone oxidation, UV irradiation, or chemical grafting to introduce polar groups (like hydroxyl, carbonyl, or carboxyl) on the polymer surface [100,102]. For instance, plasma treatment in air or O_2_ can etch and oxidize the surface of PE/PP films, adding oxygen functionalities. This makes the surface more hydrophilic, which can improve wettability and allow microbes to attach and colonize the plastic more easily [100,101]. Once microbes form a biofilm, they may secrete acids or enzymes that further attack the polymer (at least the oxidized layer) [100]. Plasma or corona treatment is already used industrially to promote ink adhesion on polyolefins, and applying it more intensely can produce a thin oxidized layer [102]. However, by itself, this layer is extremely thin (nanometers), so total conversion of the polymer is still limited by the unoxidized interior [100,101].

To extend chemical modification deeper, some studies have used chemical oxidation—for example, treating polyethylene in a solution of nitric acid or potassium permanganate to deliberately oxidize it [101]. This is not practically scalable for consumer products (and raises environmental concerns of their own) but serves as a model: heavily oxidized PE can become biodegradable to some extent, as it starts resembling a polyacid or polyester [100,101]. Graft copolymerization is another tactic, where one grafts a degradable polymer onto the surface of polyolefin items [103]. For instance, grafting poly(ε-caprolactone) or polyesters onto PE backbone (often by radiation grafting or reactive extrusion) can create a comb-like structure where the polyolefin main chain has biodegradable side chains [100,103]. The idea is microbes degrade the side chains and perhaps break the main chain apart in the process. Grafting of hydrophilic polymers (like poly(vinyl alcohol), PVA, or poly(ethylene oxide), PEO) onto PE has also been carried out to increase hydrophilicity and swelling, which in turn can facilitate enzymatic access [100,101].

Radiation (gamma or e-beam) can also induce crosslinks and chain scissions in PE/PP; interestingly, if performed in air at a controlled dose, it creates oxidized crosslinked networks that are more brittle and can fragment easier in soil [103]. But radiation can embrittle the material to the point of losing utility before use, so it is a fine balance.

One novel surface approach is biofilm inoculation: seeding plastics with certain biodegradation-promoting microbes or enzymes on their surface. For example, impregnating a plastic surface with spores of Penicillium fungus known to degrade plastics, under the notion that when disposed, those spores germinate and attack the plastic [99]. This is speculative and not widely practiced, but conceptually interesting.

In summary, surface and chemical treatments can accelerate the initial phase of degradation (making the material wettable, oxidized, and microbe-friendly), which is often the hardest step for polyolefins [100,103]. A multi-step treatment might be envisioned: e.g., produce a PP film, give it a pro-oxidant additive, and perform a plasma treatment. The plasma creates immediate surface oxidation, and the additive continues the oxidation deeper once triggered by UV, resulting in a material that fragments and weathers much faster than neat PP [103]. Care must be taken that these modifications do not unduly compromise the material during its intended service life [100,101].

## 14. Enzyme-Assisted and Biological Approaches

Taking inspiration from nature, researchers are exploring ways to involve enzymes or microbes directly in breaking down PE and PP [99,104]. One approach is to embed or coat the polymer with enzymes that can degrade it. As mentioned earlier, the discovery of waxworm saliva enzymes that oxidize PE is a breakthrough [99]. While we cannot yet produce those enzymes cheaply at scale, one could imagine in the future stabilizing such enzymes in a polymer matrix [99].

In fact, a very recent success story is in PLA: Guicherd et al. [105] reported a PLA film with a tiny amount of an engineered enzyme dispersed in it that can self-degrade the plastic in home compost conditions. They used a thermostable PLA-depolymerase enzyme, mixed it into a PLA-PCL matrix (via masterbatch) at 0.02%, and found the film fully disintegrated in ~20 weeks at 30 °C home compost—meeting compostability standards [104]. Importantly, the enzyme remained inactive during the plastic’s use (the plastic could be stored normally), but once in moist compost, it catalyzed depolymerization of PLA. This concept of enzyme-embedded plastics is now proven for polyesters, and researchers are looking at whether similar could be achieved for polyolefins [99,104].

The challenge is that no known enzymes vigorously depolymerize PE/PP on their own [99]. The waxworm enzymes are oxidases that perform the initial oxidation; conceivably, one could embed those in a plastic along with a small oxidant to continuously produce radicals that break the polymer. Another idea is enzyme immobilization: attaching enzymes like laccase or peroxidase onto a polymer surface to help initiate breakdown when triggered by moisture or appropriate co-substrates [99,106]. For PE and PP, suitable enzymes are still being sought; candidates include certain laccases, manganese peroxidases, alkane hydroxylases, or recently discovered alkane-degrading enzymes from bacteria [99].

A related bio-approach is using genetically engineered microorganisms. Scientists are exploring synthetic biology routes to either enhance known plastic-degrading microbes or create new metabolic pathways [99]. Waxworm gut bacteria that can consume PE (like Achromobacter and Klebsiella strains found in waxworms) are being genetically studied to identify the genes involved [99]. Another concept is a two-stage degradation system: first, use an engineered fungus or algae to secrete oxidative agents that make microplastics more palatable, and then have bacteria metabolize the resulting compounds [99].

A simpler near-term method is to incorporate organic materials that carry their own degrading biology. An example: some companies have additives claimed to be “enzymatic additives”, essentially organic compounds (like proteins or peptides, possibly from soy or other sources) that purportedly attract microbes to the plastic and stimulate biofilm formation that leads to degradation [99]. Independent studies on their efficacy have had mixed results—some show increased microbial growth on plastic samples with these additives, but not necessarily significantly faster mineralization [99]. Nonetheless, it underscores the idea of recruiting nature’s agents to do what they do best.

## 15. Photocatalytic and Nanoparticle Additives

Lastly, a cutting-edge strategy is adding photocatalysts or other nano-additives to assist in polymer breakdown [107,108]. Semiconducting nanoparticles like titanium dioxide (TiO_2_) or zinc oxide (ZnO) can generate reactive oxygen species when exposed to UV light [107,108]. If embedded in plastic, they can catalyze oxidative degradation under sunlight (a principle used in certain self-cleaning materials) [108]. The radicals produced by excited TiO_2_ can attack C–H bonds in PE, initiating chain scission similarly to metal stearates [109]. This occurs with the benefit that TiO_2_ itself is not consumed and is non-toxic, though it remains in the environment as mineral [109].

A concern is that these particles might detach and become free nanoparticles in the environment, so surface modification of nanoparticles to tether them in the polymer is investigated [108]. Pro-oxidant clay nanocomposites are another variant: incorporating clays intercalated with transition metal ions—these clays both weaken the polymer matrix and catalyze oxidation [107].

**Figure 16:**
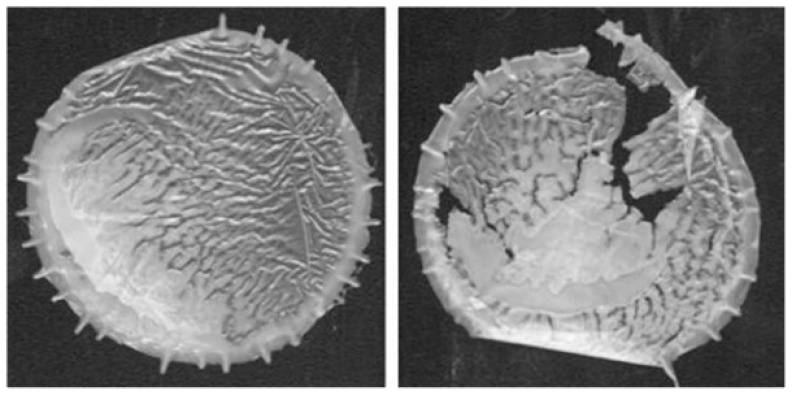
Polymer film before and after exposure to UV light [15]. Licensed under CC BY 4.0.

Additionally, there is research on UV-absorbing additives that are not stabilizers but converters of UV to chemical energy that breaks bonds [107]. For example, certain photosensitizers (like anthraquinone derivatives) can be blended into a polymer, and under UV, they produce singlet oxygen, which then attacks the polymer. These can be more precisely triggered by specific wavelengths (possibly even a laser or lamp post-use, as a way to “activate” degradation on demand) [107] (Figure 16).

Nano–bio hybrids are an intriguing concept too: e.g., immobilizing oxidative enzymes on silica or magnetic nanoparticles and dispersing those in the polymer. The particle protects the enzyme during melt processing, and once the product is out in the environment, the enzyme on the particle’s surface can work on the polymer matrix. This intersects with the enzyme embedding idea but with a focus on nano-carriers for the enzymes [107,108] (Table 3).

All the above methods aim to make inert polyolefins more reactive in the environment. Some are already applied (oxo-additives in agricultural films, etc.), whereas others are at research stage (enzyme-embedded plastics). It is likely that a combination of approaches will be needed—for instance, a future polyethylene film might contain a small fraction of ester-linked comonomer and a pro-oxidant additive, and perhaps a trace of a catalytic particle, each contributing to a quicker degradation sequence after the product’s useful life. The ideal end goal is a polyolefin that serves its function during use, and then, once discarded into the appropriate environment (compost, soil), it first oxidizes (loses mechanical strength), then is consumed by microbes, leaving no persistent residues or toxic by-products. The next section discusses the broader context and implementation challenges for these solutions (Table 4).

## 16. Challenges, Future Directions, and Circular Integration

Designing degradable plastics means trying to balance performance, cost, and environmental benefit. Several overarching challenges remain before degradable single-use plastics (whether new materials or modified PP/PE) can be widely implemented:

Performance Trade-offs: Many biodegradable alternatives and modified plastics have inferior material properties compared to conventional PP/PE. For example, PLA is brittle and has a low heat distortion temperature, starch-based materials are moisture-sensitive, and adding pro-degradant additives can reduce polymer strength and shelf life [43,123]. There is often a direct trade-off between durability and degradability. The very property that makes plastic last in use (resistance to heat, oxidation, mechanical stress) makes it persist in the environment [124]. Future development needs to tailor materials for the required service life—e.g., a straw or take-out container only needs stability for a few weeks or months, not decades. Controlled-life plastics are an emerging concept: plastics engineered to stay intact during use but actively degrade upon disposal (triggered by UV, moisture, or a biological trigger). The enzyme-embedded PLA is a good example of this concept working (stable in usage, rapidly degrading in compost). Extending this idea to robust polymers is a research frontier [43].

Cost and Scalability: Conventional plastics benefit from massive economies of scale and decades of optimization. Biodegradable alternatives often require more complex production (fermentation for biopolymers, purification, specialized polymerization). Although prices are coming down, materials like PLA, PBAT, and PHA remain costlier than PE/PP [43,124]. As of mid-2020s, PLA was around USD 2000/ton and PBAT similar, versus USD 1000–1200/ton for PE. Scaling up production (new biorefineries, chemical plants) is ongoing—for instance, PLA plants in Thailand and PBAT plants in Europe/Asia ramping up capacity. Economic incentives or policy (e.g., carbon credits, single-use plastic bans) may be needed in the interim to spur adoption despite higher costs. Additionally, feedstock sustainability must be managed: using food crops for bioplastics raises concern. There is a push toward using non-food biomass (cellulosic sources, waste oils, captured CO_2_ via industrial biotechnology) to produce biopolymers sustainably, which could also stabilize feedstock costs [43].

End-of-Life Management: A major challenge is ensuring that these degradable plastics end up in conditions where they actually degrade. If compostable plastic bags are not collected or if mixed into regular waste, they may just go to landfill where they will not degrade, or may generate methane anaerobically [123,125]. Worse, if biodegradable and non-biodegradable plastics are mixed, it can contaminate recycling streams (e.g., PLA contaminating PET recycling). Proper labeling and consumer education are critical. Some jurisdictions use certifications like “OK Compost Home,” “Marine OK,” or “ASTM D6400 Compostable” to guide disposal. Integration with the circular economy means designing materials that align with either the biological cycle (compostable) or the technical cycle (recyclable). Biodegradable plastics mostly align with the biological cycle, but we require the infrastructure (composting or digestion) to process them at the end of life [126]. There is also opportunity to combine recycling and biodegradation: durable plastics recycled mechanically, and biodegradable materials for hard-to-collect or contaminated items (films, food service ware).

A key practical hurdle is collection and sorting: even if a plastic product is labeled “compostable” or “oxo-degradable,” it only achieves its intended end-of-life pathway if it enters the correct stream. For example, compostable bags diverted into general landfill show little aerobic breakdown and may instead generate methane under anaerobic conditions. Many municipalities report that compostable plastics are not accepted by their organic’s programs, leading to disposal as residual waste. In Canada, one regulatory consultation noted that compostable plastics “are typically screened out by organics processing facilities and sent to landfill due to confusion and contamination with other types of plastics.” [122]. Without effective separate collection systems or clear signage and consumer education, materials intended for the “biological cycle” end up in the technical cycle (landfill or incineration), undermining their value.

A second critical challenge is cross-contamination of recycling streams. When biodegradable, compostable, or bio-based plastics inadvertently enter mechanical recycling of conventional polymers, they can impair product quality. For instance, the polymer polylactic acid (PLA), often used in compostable packaging or 3D-printing waste, if mixed into a stream of polyethylene terephthalate (PET) for recycling, may cause hazing, yellowing, and reduced melt strength. Studies report that PLA contamination levels as low as ~1000 ppm (0.1%) in PET recycling may already degrade mechanical or optical properties of rPET [127]. In a report from the UK consultancy Eunomia Research & Consulting Ltd. From Bristol, UK, operators of material-recovery facilities flagged that bioplastics “are not considered compatible with most established mechanical recycling processes” and that “if PLA is not sorted and removed … it acts as a contaminant and will have a detrimental impact …” [128].

In a study of bioplastics in Southeast Asia, the report found that while materials such as PLA, PBS (polybutylene succinate), or TPS (thermoplastic starch) have theoretically multiple end-of-life routes (mechanical recycling, composting, anaerobic digestion), in practice, the dominant outcome in Thailand was landfill or open-dumping, because the required segregation, sorting infrastructure, and dedicated streams did not exist. The authors note, “Biodegradable bioplastics … cannot be mixed in with traditional mechanical recycling processes with petrochemical plastics.” [129].

A study from Wageningen University & Research (in cooperation with industry) examined the effect of PLA trays entering the PET bottle recycling stream. The results suggested that current near-infrared (NIR) sorting technology can maintain a PLA contamination below ~1%, and at that level observed, little adverse effect on recycled PET appearance or thermal properties. The authors concluded that “in case the sorting and recycling facilities maintain their current careful operation, no negative impact of PLA on PET recycling can be foreseen.” [70]. This suggests that with robust sorting and low contamination levels, integration is possible, but this requires investment, effective separation, and traceability of grades.

Incomplete Degradation and Ecotoxicity: Even when plastics are labeled biodegradable, complete degradation is not always achieved, and intermediate oligomers may persist. For example, starch–polyolefin blends may leave behind polyolefin residues that are slow to degrade. There is also concern about additives, that metal residues from pro-oxidants could affect soil health, and that acidic breakdown products could alter pH. Standards generally require ecotoxicity testing (e.g., plant germination assays). Ahsan et al. [93] traced PBAT degradation in soil and identified microbial pathways and intermediate metabolites, demonstrating that some residues may have ecological impact without full mineralization. Continuing research should monitor real-world degradation outcomes across different ecosystems, including microbial community shifts and aquatic toxicity [93,126].

Regulations and Public Perception: Regulatory frameworks are evolving. The EU has banned oxo-degradable plastics that do not meet stringent biodegradability criteria often required by standards [43,123]. Countries like France restrict labeling terms such as “biodegradable” unless standards are met. This is critical to prevent greenwashing. As standards tighten, companies must provide robust evidence (lab data, field trials) for biodegradability claims. Public perception is also a challenge: consumers may believe biodegradable items will degrade quickly in all contexts, but without proper conditions, they may persist for months or years. Education on disposal and realistic expectations is key to proper implementation.

**Figure 17:**
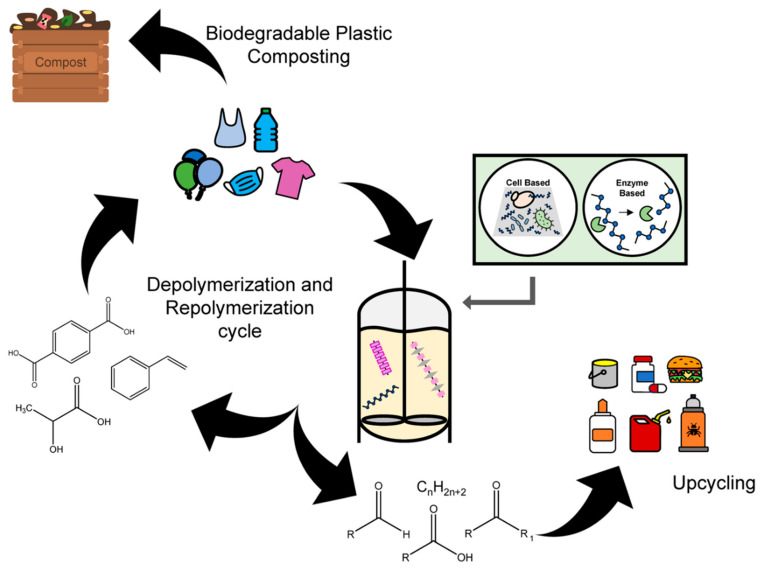
Typical lifecycle for plastic waste [130]. Licensed under CC BY 4.0.

Recycling vs. Biodegradability: There is frequently a tension between recyclability and biodegradability. Additives that promote degradation can interfere with recyclability (e.g., reduce thermal stability during re-melting). From a circular economic standpoint, materials should ideally be channeled into either the recycling loop or the biological degradation loop, not both. Durable PP/PE should remain in the technical loop (preferably mechanically recycled), while biodegradable materials are reserved for use cases where environmental leakage is likely or collection impractical. Meanwhile, chemical recycling approaches, such as pyrolysis or gasification of mixed or soiled polyolefins, offer potential alternatives to complete biodegradability, reducing reliance on environmental degradation. The reality is likely to be hybrid systems combining mechanical recycling, chemical recycling, and biodegradable solutions across various applications [43,126] (Figure 17).

## 17. Future Directions

Research is actively proceeding on multiple fronts. A promising direction is bio-based polyolefins (like bio-PE made from sugarcane ethanol) combined with new degradability features [43,105]. For instance, developing a PE that is from renewable resources and has cleavable linkages would tick many boxes. Another area is smart additives that respond to triggers, for example, a time-temperature dependent degrader that only kicks in after a product’s expected lifespan. Synthetic biology might yield more efficient plastic-degrading enzymes or even living hybrid materials (imagine packaging material that has dormant microbes or enzymes that activate upon disposal to self-degrade) [19,105]. Nanotechnology could produce materials that change structure after use (like programmed degradation via nanocapsules releasing acids or enzymes). Additionally, lifecycle analysis (LCA) and systems thinking will guide what solutions make sense environmentally [43,105]. It could turn out that for some uses, improving recycling yields more benefit than switching to a biodegradable plastic, whereas for others (thin films, multi-layer sachets), designing them to biodegrade might be better since those are hard to recycle.

From a policy perspective, likely there will be more mandates for compostable packaging (for food-contact disposables, etc.) in regions where organics collection is in place. Industries such as agriculture (mulch films) and fisheries (lost fishing gear) are actively interested in biodegradable options because their plastic products often end up in the environment by design or accident. We can expect more tailored products like biodegradable fishing nets (there is research on PBS/PHA blends for nets) that degrade if lost at sea, or biodegradable mulch films (already some PBAT/starch films are used commercially) that farmers can plow into soil. In the medical field, biodegradable polymers (e.g., PLLA, PCL, PHA) are used for implants and could expand as well [43,131].

A possible vision is polyolefins that are both recyclable and biodegradable; though those seem almost contradictory, clever chemistry might allow a plastic to be depolymerized (chemically or enzymatically) in a controlled setting for recycling, but also to biodegrade if it escapes into nature. For example, a polymer with cleavable bonds could be chemically broken down for recycling into monomers, and the same bonds would allow environmental breakdown if not collected [84]. This kind of dual-purpose design would truly align with circular economy ideals by covering both closed-loop reuse and open-end biodegradation as a safety net.

## 18. Proposed Research Hypotheses and Experimental Methodologies

To further advance degradable single-use plastics, a number of research directions can be pursued. Below, we outline some actionable, lab-testable hypotheses and how one might experimentally investigate them, including specific materials, degradation tests, and performance metrics. These proposals build on the current state of science discussed above:

Synergistic Oxo-Bio Additives in Polyolefins: Hypothesis: Combining a pro-oxidant additive with an enzyme-mimicking catalyst will lead to more complete biodegradation of PE/PP than a metal additive alone. Methodology: Formulate PE films with (a) a conventional metal stearate additive (e.g., 1% cobalt stearate), and (b) a dual additive: metal stearate + a small amount of a photocatalyst (e.g., 0.5% TiO_2_ nanoparticles) or an organic sensitizer. Subject both to accelerated aging (UV chamber at ~60 °C, 0.6 W/m^2^ UV intensity) for defined periods (e.g., 100, 200 h). Monitor oxidation by measuring the carbonyl index via FTIR and molecular weight via GPC. Then, take aged samples and perform biodegradation tests (ASTM D5988 soil burial, measuring CO_2_ evolution over 6 months). Metrics include rate of CO_2_ production (% biodegradation) and residual polymer weight, plus tensile strength post-aging to confirm in-use durability. The prior literature supports that photocatalysts speed up oxidation, but it remains untested whether that yields proportional microbial assimilation [107].

Enzyme–Polyolefin Composite with Waxworm Enzyme: Hypothesis: Embedding purified waxworm saliva oxidase into a polyolefin matrix (via a protective carrier) can initiate PE depolymerization under mild conditions. Methodology: Express and purify the two key enzymes identified by Sanluis-Verdes et al. [19] in a fermentation system. Immobilize them onto silica or PCL nanoparticles to improve thermal stability during processing. Incorporate 0.1–0.5% enzyme-loaded particles into LDPE via solution casting or low-temperature melt compounding (<100 °C). Produce thin films and incubate in buffered aqueous medium (pH ~ 7, 30 °C). Periodically measured oxidation products (GC-MS detection of short-chain acids/aldehydes, carbonyl index via FTIR). Then, add a microbial inoculum for respirometry (CO_2_ evolution) and compare enzyme-containing versus control films. Metrics: molecular weight changes, oxidized chemical group formation (e.g., via FTIR or XPS), and CO_2_ evolution. Controls should include heat-inactivated enzymes to confirm activity-specific effects. Demonstrating even partial oxidation of PE under ambient conditions would be significant [19,131].

Bio-based Copolymer Blends for Marine Degradation: Hypothesis: A blend of polyolefin with a PHA copolymer, compatibilized at the interface, will result in the polyolefin component degrading in marine conditions more than if it were alone. Methodology: Using reactive extrusion, create a blend of PP (or PE) with 20–30% of a medium-chain-length PHA (which is flexible and tough) and add a graft compatibilizer (e.g., maleic anhydride-grafted PP) to ensure fine dispersion. Manufacture film samples. Marine degradation test: Follow ASTM D6691 by placing samples in natural seawater-inoculated medium at 30 °C, with gentle aeration. Run for 6 months, measuring CO_2_ evolution in sealed respirometer flasks. Additionally, periodically remove samples to measure weight loss and observe surface via SEM. The PHA phase should biodegrade relatively quickly (expected within 2–3 months, known from the literature). After the PHA is gone, examine the PP residue for any signs of embrittlement or biodegradation. Possibly use gel permeation chromatography to see if PP molecular weight decreased, and FTIR to see if new oxygen-containing groups appeared (indicative of some oxidative or biological attack). Metrics: Total CO_2_ output attributable to PP (beyond what PHA alone would give, subtracting a control with pure PHA). Also, mechanical properties of the film over time (tensile test) to see if it loses integrity faster than neat PP in seawater. This would test whether the presence and degradation of PHA facilitates some biodegradation of PP in a marine-like environment. If successful, it suggests that blending polyolefins with easily biodegradable polymers and proper compatibility might help mitigate pollution in open environments like oceans.

Long-Term Soil Burial of Ester-Modified Polyethylene: Hypothesis: Polyethylene containing cleavable ester bonds (from copolymerization) will substantially mineralize in soil over extended time, whereas normal PE will not. Methodology: Synthesize an ester-containing PE (e.g., via the method of Zeng et al. [84], with perhaps 3–5 mol% ester units). Prepare pure PE and ester-PE samples (like pellets or films). Bury them in a well-controlled soil environment (large pots or soil columns kept at 25 °C with controlled moisture and aeration). Include positive controls (cellulose strips for which complete biodegradation is expected). Every 3 months, measure CO_2_ evolved from each vessel (using traps or an automated respirometer). Also, at 6, 12, 18, and 24 months, dig up some samples to analyze remaining polymer (molecular weight, any erosion). Potentially use isotope labeling (e.g., if the polymer can be made from ^13^C labeled feedstock) to trace the conversion of polymer carbon to CO_2_ unambiguously. Metrics: Percent carbon converted to CO_2_ over 1–2 years for modified vs. unmodified PE. Even a modest conversion (say 5–10% of the carbon) for the modified PE would be a remarkable result because conventional PE would be ~0% in that timeframe. This long experiment directly gauges real-world biodegradation potential of chemically modified polyolefins and would inform how effective such structural changes are when no artificial acceleration (like high-temperature composting) is present.

Mechanical Performance vs. Degradability Trade-off Study: Hypothesis: There exists an optimal loading of pro-degradant or bio-filler at which a polyolefin retains acceptable mechanical properties for use but significantly improves its degradation post-use. Methodology: Take PP as a model. Prepare a series of PP compounds with increasing additive load: e.g., 0%, 1%, 3%, 5% of a pro-degradant package (could be Mn-stearate + small % starch as a combined approach). Characterize initial mechanical properties (tensile strength, elongation, impact toughness). Then, expose all samples to a simulated life cycle: say 1 month of UV aging + thermal cycling to mimic typical outdoor litter exposure. After that, conduct biodegradation testing on the aged samples in compost or soil for 90 days. Metrics: Tensile strength vs. additive level, and biodegradation % vs. additive level. Determine the highest additive level at which properties are within, say, 80% of neat PP. See how much biodegradation that level yields compared to neat PP. This provides practical insight into how far we can push the modification without losing function. For instance, maybe 3% additive only reduces strength by 10% but doubles biodegradation rate—a good trade-off, whereas 5% additive might compromise product use (e.g., brittle failure), which is not acceptable. Such results would help guide industrial adoption by highlighting the “sweet spot” of modification intensity.

Enzymatic Recycling Feasibility for Bioplastics: Hypothesis: Biodegradable polyesters (PLA, PBS, PCL, etc.) can be efficiently depolymerized by enzymes, allowing their recovery and reuse, which can complement their biodegradation. Methodology: Although not directly about polyolefins, this has relevance for the circular economic integration. Take post-consumer PLA and PBAT waste samples (e.g., compostable cups, bags) and apply cocktails of enzymes: PLA-depolymerase for PLA, lipases for PBAT (which have ester bonds). Measure depolymerization in a bioreactor (monomer or oligomer release, via HPLC). Also, test if mixed streams (PLA + PBAT together) can be simultaneously depolymerized by adding multiple enzymes. Metrics: Percent of plastic converted to soluble monomers (lactic acid, succinic acid, terephthalate, etc.) in 48 h at optimal conditions. This addresses an important question: while these materials are biodegradable, can we also recycle them biologically rather than composting (which just yields CO_2_ and waste)? A positive result would suggest that in the future, enzyme reactors at recycling facilities could recapture monomers from biodegradable plastics to make new polymer—combining bio-circular approaches.

Each of these proposed experiments is grounded in current scientific understanding and addresses open questions. They also provide training opportunities in interdisciplinary methods—from polymer chemistry (synthesis, characterization) to microbiology (biodegradation assays) and materials testing (mechanical, environmental aging). A research program built around these hypotheses would yield valuable data on how to realistically create plastics that meet use requirements yet do not burden the environment long after their use.

Finally, it is worth noting that any promising lab result should be followed by pilot-scale trials and real-world testing. For instance, if a new additive package for PE shows 50% biodegradation in lab compost tests, the next step is to make actual prototype bags, test their shelf life, and then compost them in an industrial facility and analyze residuals. Similarly, field litter tests (placing samples in natural settings like a marine environment or soil under outdoor conditions) are essential to validate performance outside the controlled lab. Only through such comprehensive evaluation can we ensure that the solutions we develop for degradable plastics are both effective and responsible.

## 19. Conclusions

Creating degradable single-use plastics involves chemistry, biology, engineering, and environmental science [43,124]. Polypropylene and polyethylene pose a steep challenge due to their stubbornly inert nature. Yet, as this review has detailed, multiple avenues are being pursued to reduce their persistence. Biodegradable alternatives like PLA, PHAs, and starch-based materials offer immediate replacements in certain applications, though each comes with limitations in strength, cost, or required composting infrastructure [43,123]. Innovative modifications to traditional plastics such as adding oxidative additives to synthesizing cleavable polymer backbones seek to change how we deal with plastic lifecycles [43,84].

A key theme is that degradation is context-dependent: what readily biodegrades in an industrial composter may persist in the ocean, and what fragments under intense sunlight may lie almost inert in a dark landfill. Therefore, a material’s environmental compatibility must be evaluated under the range of plausible end-of-life scenarios [126]. The development of international standards and more rigorous field testing is helping ensure that “biodegradable” claims reflect real outcomes [123]. The ultimate goal is not simply to make plastics disappear faster, but to ensure they break down into environmentally benign constituents without leaving behind a legacy of microplastic or toxic residue [93]. This must be achieved while still delivering the performance and safety consumers expect from plastic products, and at a reasonable cost and carbon footprint [43,124].

In the broader circular economy vision, degradable plastics play an important supporting role but not a solitary one. They work with improved product design (to reduce unnecessary single-use items), better waste collection and recycling systems, and public behavior shifts (reusing items, separating organics, etc.) [43,126]. For instance, compostable packaging makes sense only if coupled with food waste collection and composting facilities. Likewise, integrating enzyme-triggered plastics might pair with industrial composters or digesters to recover value as biogas or compost. In some cases, durable recyclable plastics may be environmentally preferable (if we can recapture them), underscoring that “degradable” is not synonymous with “sustainable” unless applied thoughtfully [43,126].

Looking forward, there are many different directions the field can advance in. Advances in catalysis, synthetic biology, and materials science are converging; we might see bespoke enzymes that can chew up polyethylene, or polymers that are programmed to self-destruct after a certain period [19,105]. Techno-economic analysis will be essential too—identifying which solutions offer the biggest environmental gain for the least economic and energy cost [43,124]. Perhaps the most realistic near-term implementations will be hybrid approaches: e.g., a mainly bioplastic product with a tiny conventional plastic component for performance, where that component is designed to degrade eventually, or a mostly recyclable plastic product that has a biodegradable coating to deal with unrecyclable contamination.

In summary, solving the plastic pollution problem requires rethinking materials from design to disposal. Degradable plastics and polyolefin modifications are critical pieces of that puzzle [43,126]. The literature reviewed here shows significant progress in understanding degradation mechanisms, creating new biodegradable materials, and even tweaking polyolefins to be less everlasting. Challenges remain, but the trajectory is set: future plastics will not be “forever” plastics. By continuing to refine these materials and strategies, we can move toward plastics that serve society during their useful life and gracefully reintegrate into the natural carbon cycle thereafter. This will be a major step toward alleviating the environmental burden of single-use plastics while maintaining the benefits they have brought to modern life [43,124].

## Figures and Tables

**Table 1:** Comparison of conventional polyolefins and selected biodegradable alternatives.

Polymer	Primary Feedstock	Biodegradability	Typical Degradation Environment/Time	Notable Properties and Uses	Production Scale (Approx.)
Polyethylene (PE)	Petroleum (fossil)	No (non-biodegradable)	Persists in all environments; fragments under UV over years	Tough, flexible, moisture barrier; ubiquitous in packaging (bags, films, bottles)	>100 million tons/yr (for PE)
Polypropylene (PP)	Petroleum (fossil)	No (non-biodegradable)	Persists long-term; some oxidation under UV (faster than PE) but negligible biodegradation	Rigid, versatile (containers, caps, fibers); high chemical resistance	~70 million tons/yr
Polylactic Acid (PLA)	Corn or sugar (dextrose)	Yes (industrial compost)	~90% biodegrades in 2–4 months at 58 °C compost; slow in soil (years) and negligible in marine	Clear or opaque; high strength but low T_g (60 °C); used in food packaging, utensils, 3D printing	~0.2–0.3 million tons/yr (2020s)
Polyhydroxyalkanoate (PHA) (e.g., PHB/PHBV)	Sugars/oils via fermentation	Yes (broadly biodegradable)	Biodegrades in many environments (compost ~months, soil months–year, marine months–year)	Brittle (PHB homopolymer), improved with copolymers; used in packaging, agricultural films, medical implants	<0.05 million tons/yr (nascent but growing)
Polybutylene Succinate (PBS)/PBAT	Glucose (bio succinic) or fossil (terephthalate)	Yes (compost, soil)	Compost in <6 months; in soil ~year; limited data in marine (PBAT partial)	Flexible, tear-resistant (especially PBAT); often blended with PLA or starch (e.g., compostable bags)	~0.1–0.2 million tons/yr (PBAT)
Starch-based Plastic (TPS or starch blends)	Corn, potato, etc., starch	Yes (rapidly)	Readily biodegrades in soil/compost (weeks to months, depending on formulation)	Low-cost, can be edible; poor water resistance, moderate strength; used in packing peanuts, bags (with blends)	– (blended in many products, starch is cheap filler)
Cellulose (Cellophane)	Wood pulp or cotton	Yes (rapidly)	Soil/compost in weeks–months (uncoated cellulose film)	Very low gas permeability, non-static; but hydrophilic and tears when dry; used in specialty packaging, tubing	Limited (<0.01 Mt/yr, niche)
Cellulose Acetate	Wood pulp (modified)	Yes (if low DS)	Low substitution (DS < 2) degrades in months; high DS (e.g., cigarette filters) takes years	Tough, glossy, used in fibers, filters, tool handles; can be formulated to biodegrade by adjusting acetylation	Moderate (especially in fiber form for textiles)
Chitosan	Crab/shrimp shells (chitin)	Yes (slow in soil)	Biodegradable (enzymatically by some microbes); thin films in soil months–year	Antimicrobial, edible, but soluble in acid and mechanically weak; used in edible coatings, medical films	Very limited (lab-scale applications)
Protein-based (e.g., casein, soy)	Milk protein, soybeans	Yes (enzymatically)	Readily broken down by microbes (weeks) if wet; durable if kept dry (not environmental)	Edible/biocompatible; poor water resistance; historical use (casein plastic artifacts), now in edible packaging research	Very limited (not industrially produced in bulk)

Notes: The data above are generalized; actual degradation times vary with thickness and conditions. “Industrial compostable” plastics like PLA, PBS, and PBAT require the high heat and microbial density of industrial facilities to degrade efficiently. PHA and starch materials can biodegrade under broader conditions (including marine for PHA). Cost-wise, most bioplastics (PLA, PHA, PBAT) are currently 2–5 times more expensive than commodity PE/PP (which are ~USD 1000–1500/ton), although prices are coming down as production scales up. Lifecycle assessments (LCAs) indicate that bio-based biodegradable plastics can reduce fossil fuel use and greenhouse emissions, but only if they are composted or biodigested at end of life; if landfilled or littered, their environmental advantage diminishes and could even reverse in the case of anaerobic methane release or slow degradation.

**Table 2:** Comparative production capacities, approximate market costs, and key mechanical properties of major commodities and biodegradable polymers.

Polymer	Production Capacity (Mt·yr^−1^)	Cost (USD·t^−1^)	Key Properties (Concise)	Typical Uses
Polypropylene (PP)	~70–100 Mt/yr (global, recent years) [70]	~600–1400 USD/ton (market/industrial price ranges reported in datasets and market syntheses; varies with feedstock and region) [70]	Lightweight polyolefin, good chemical resistance, semi-crystalline, good stiffness (can be tough), weldable, recyclable (thermoplastic) [70]	Packaging (rigid and flexible), automotive (interiors, bumpers), fibers, consumer goods, medical disposables [70]
Polyethylene (PE)	~110–140 Mt/yr (global, combined PE grades) [70]	~700–1400 USD/ton (varies by grade, region and feedstock) [70]	Versatile polyolefin family; ranges from flexible (LDPE, LLDPE) to rigid (HDPE); excellent chemical resistance, low permeability (grade dependent), good processability [70]	Film and packaging, bottles (HDPE), pipes, geomembranes, containers, cable insulation [70]
Polylactic acid (PLA)	~0.3–0.8 Mt/yr in the literature, and bioplastics capacity reports give values in the few 100 kt range (growing) [46]	~1000–3500 USD/ton (TEAs and market pricing show wide range depending on feedstock, scale, and region [71]	Semi-crystalline (depending on stereochemistry), bio-based (from fermentable sugars), good stiffness and clarity, brittle vs. some petro polymers (can be modified), industrially compostable under proper conditions [72]	Packaging (rigid cups, trays, films), fibers (textiles), 3D printing (filaments), disposable cutlery/containers, biomedical resorbable implants [72]
Polyhydroxyalkanoates (PHA)	~100 kt/yr current global bioplastic PHA capacity—order-of-magnitude; expanding but still small vs. commodity plastics [73]	~4000–6000 USD/ton [74]	Microbially produced polyesters (e.g., PHB, PHBV): biodegradable, good barrier to oxygen (varies), brittle in some homopolymers (copolymers improve toughness), processable thermoplastically after extraction/purification [75]	Compostable packaging, mulch films, agricultural films, specialty medical applications, additives/blends to improve biodegradability [75]
Poly(butylene adipate-co-terephthalate) (PBAT)	~(80–150 kt/yr) [73]	~3500–4500 USD/ton [76]	Aliphatic–aromatic co-polyester; flexible, good elongation and toughness, biodegradable under industrial composting (when blended/under enzyme/microbial action), relatively low Tg [77]	Flexible films (compostable bags), mulch films, blend component to impart flexibility to compostable formulations (e.g., PLA/PBAT blends) [77]
Poly(ε-caprolactone) (PCL)	~55 kt/yr [78]	~6000–8000 USD/ton [79]	Aliphatic, low Tg (rubbery at room temp. depending on Mw), biodegradable (enzymatic hydrolysis), excellent blend/compatibilizer/solvent-casting behavior; used widely in biomedical applications due to biocompatibility [80]	Specialty biodegradable formulations, biomedical devices (drug delivery, scaffolds), blending/compatibilizer in biodegradable formulations, hot-melt adhesives [80]

**Table 3:** Strategies for enhancing polyolefin degradability.

Modification Strategy	How It Works	Example Outcome (Ref.)	Remarks/Challenges
Copolymers with cleavable bonds	Insert hydrolysable or weak bonds (ester, ketone) in polymer backbone	PE copolymerized with 5% cyclic ketene acetal -> degrades into oligomers in base.	Maintains PE-like properties if low comonomer; need sufficient cleavable units to aid biodegradation.
Pro-degradant metal additives	Catalyze oxidation (radical formation)	PP + 1% Mn/Ca stearate additive: complete embrittlement in 8 weeks of sunlight; ~60% biodegradation in 2 years in soil	Ensures fragmentation, full mineralization still slow. Regulatory acceptance issues (microplastics concerns).
Blending with biodegradable polymer	Creates composite where one phase degrades quickly, leaving polyolefin exposed	PE + 15% starch blend: starch consumed in weeks, PE breaks into micro-fragments more readily.	Residual PE still persists; mechanical properties may drop if blend is high in bio-filler.
Surface oxidation (plasma, etc.)	Introduces polar groups, increases wettability and roughness	Plasma-treated PE film: 5× higher surface oxygen content, allowed biofilm formation of *Pseudomonas* in 2 days vs. 7 days for untreated.	Only surface is modified; effect is mainly on initial colonization. Bulk remains unchanged.
Enzyme embedding/coating	Enzymes directly attack polymer (if possible) or catalyze breakdown steps	Engineered cutinase in PLA film -> full disintegration in 5–6 months at 30 °C. Waxworm oxidase on PE surface -> initiated oxidation at room T.	Enzyme stability is a concern; need enzymes active on polyolefins (still under exploration). Cost of enzymes can be high.
Photocatalyst/sensitizer addition	Particles generate reactive oxygen under light, accelerating polymer oxidation	LDPE with TiO_2_ nanoparticles exposed to UV had 2× carbonyl index increases vs. neat LDPE, indicating faster oxidation.	Requires UV exposure (not helpful in dark landfill); nanoparticles must be well dispersed; ensure they do not introduce toxicity.
Bio-augmentation (microbes)	Use of microbes engineered or selected to consume polymer fragments	Waxworm gut bacteria added to soil with PE film showed ~10% weight loss of film in 1 month vs. ~2% in sterile control.	Hard to control in open environments; works best in managed waste treatment (bioreactors).

**Table 4:** Comparative degradation behavior of selected polymers across marine, soil, industrial composting, and anaerobic (landfill) environments.

Polymer	Marine (Seawater)	Soil (Temperate)	Industrial Compost (Thermophilic, ~>50–60 °C)	Anaerobic/Landfill
PP/PE (polyolefins)	Very slow/negligible under natural marine conditions, persistence for years to centuries; little biodegradation [110]	Very slow, years to decades; essentially persistent in typical soils [110]	Do not reliably biodegrade in standard composting; physical fragmentation possible but mineralization negligible [110]	Very slow; in anaerobic landfills, bulk polyolefins persist for decades (little biotransformation) [111]
PLA	Very slow in cold marine environments; limited mineralization in seawater, months → years (lab/field vary) [112]	Slow in ambient soils (months → years), often limited without elevated temperatures/adapted microbes [112]	Relatively fast under industrial composting (thermophilic): weeks → few months to high (>70–90%) mineralization under standard industrial conditions. But home-compost or cool compost much slower [112]	Limited anaerobic degradation in typical landfills/AD—PLA often does not fully biodegrade in anaerobic digesters/landfills on timeframes used; conversion depends on temp. and pretreatment [112]
PHA (e.g., PHB, PHBV)	Generally fast relative to many other plastics, many PHAs show substantial biodegradation in seawater (weeks → months) in lab/mesocosm studies; marine PHA-degrading microbes are widespread [113]	Fast to moderate, weeks → months depending on polymer composition and soil microbiota; mineralization often high in soils [113]	Fast in industrial composting, weeks → months with substantial mineralization [113]	Variable: some PHAs can be degraded anaerobically (methanogenic conditions) but rates depend on polymer type/MW; studies report measurable degradation under anaerobic conditions but outcomes are variable [112]
PBAT	Slow to moderate in marine environments (many studies show limited marine biodegradation; fragmentation possible) [114]	Slow → moderate in soils (months to >1 year depending on conditions) [114]	Moderate in industrial composting (months), PBAT is designed to be compostable in many formulations when blended appropriately [115]	Slow/limited in anaerobic landfills, biodegradation under anaerobic conditions is generally poor relative to aerobic composting [115]
PCL	Variable, some marine microbes degrade PCL, lab studies show marine isolates can attack PCL; timescales often months under favorable conditions [116,117]	Slow → moderate in soil (months to >1 year depending on temp and microbes) [112]	Moderate in compost (weeks → months) under warm conditions; blends and low-MW PCL degrade faster [112]	Variable/generally slow under typical landfill anaerobic conditions; some anaerobic degradation reported under specific conditions [117]
PBS	Some evidence of marine biodegradation (low-MW PBS or specific formulations degrade faster); marine enzymes reported to degrade PBS. Timescales: months (lab) to >1 year (field) [118]	Moderate in soil (months). Recent isotope-tracing work shows measurable mineralization and improved kinetic understanding [119]	Moderate → fast in industrial composting (weeks → months) depending on MW [120]	Variable/generally slow in anaerobic landfills; some hydrolysis can occur but full mineralization is slower than in aerobic compost [117]
Cellulose and cellulose acetate	Cellulose: fast (weeks → months) where accessible and colonized by microbes; cellulose acetate: slower than native cellulose (acetylation reduces biodegradability), but some biodegradation occurs over months → years depending on DA and environment [121]	Cellulose: fast in soil (weeks → months); CA: slower but deacetylation in soil enables biodegradation over months → years [121]	Cellulose: fast in compost (days → weeks); CA: moderate (weeks → months) depending on degree of substitution and microbial access [121]	Cellulose: can be degraded anaerobically (e.g., in landfills); CA: slower but can undergo hydrolysis/biodegradation under some anaerobic conditions, timescales variable [121]
Chitosan (films)	Moderate in marine (biologically active; chitosan is biodegradable), weeks → months depending on form [122]	Moderate → fast in soil (weeks → months), chitosan is a readily biodegradable polysaccharide in many environments [122]	Fast in compost (weeks) under aerobic conditions [122]	Variable/can be degraded anaerobically (landfills/AD), but rates depend on conditions; expect slower rates than aerobic compost [122]
TPS (thermoplastic starch blends)	Often faster than synthetic polyesters; in lab marine tests, some TPS formulations show measurable degradation in months (but depends on blend with PBAT/PBS, etc.) [7]	Fast in soil (weeks → months), native starch is easily biodegraded; blends slowly depending on synthetic fraction [7]	Fast in industrial composting (days → weeks to months), especially for high-starch formulations [7]	Variable: starch fractions can be anaerobically digested (biogas) in AD systems, but blends with non-biodegradable copolymers reduce overall biodegradation in landfills [7]

## Data Availability

No new data were created or analyzed in this study. Data sharing is not applicable.
